# Translational Control of UIS4 Protein of the Host-Parasite Interface Is Mediated by the RNA Binding Protein Puf2 in *Plasmodium berghei* Sporozoites

**DOI:** 10.1371/journal.pone.0147940

**Published:** 2016-01-25

**Authors:** Patrícia A. G. C. Silva, Ana Guerreiro, Jorge M. Santos, Joanna A. M. Braks, Chris J. Janse, Gunnar R. Mair

**Affiliations:** 1 Instituto de Medicina Molecular, Faculdade de Medicina da Universidade de Lisboa, Av. Prof. Egas Moniz, 1649–028, Lisbon, Portugal; 2 Department of Parasitology, 3, Leiden, The Netherlands; 3 Parasitology, Department of Infectious Diseases, University of Heidelberg Medical School, Im Neuenheimer Feld 324, 69120, Heidelberg, Germany; Université Pierre et Marie Curie, FRANCE

## Abstract

UIS4 is a key protein component of the host-parasite interface in the liver stage of the rodent malaria parasite *Plasmodium berghei* and required for parasite survival after invasion. In the infectious sporozoite, UIS4 protein has variably been shown to be translated but also been reported to be translationally repressed. Here we show that *uis4* mRNA translation is regulated by the *P*. *berghei* RNA binding protein Pumilio-2 (PbPuf2 or Puf2 from here on forward) in infectious salivary gland sporozoites in the mosquito vector. Using RNA immunoprecipitation we show that *uis4* mRNA is bound by Puf2 in salivary gland sporozoites. In the absence of Puf2, *uis4* mRNA translation is de-regulated and UIS4 protein expression upregulated in salivary gland sporozoites. Here, using RNA immunoprecipitation, we reveal the first Puf2-regulated mRNA in this parasite.

## Introduction

RNA binding proteins play a key role in the temporal and spatial regulation of protein expression. In the rodent malaria parasite *Plasmodium berghei*—a model for the human *P*. *falciparum* parasite—post-transcriptional gene regulatory mechanisms specifically affect protein translation during the transmission of the parasite between the mosquito vector and a mammalian host which co-insides with major developmental changes [1–8]. This for example includes RNA helicase DOZI and CITH-mediated translational repression in the intra-erythrocytic female gametocyte prior to uptake during a mosquito blood meal [2, 3, 8], through mRNA binding at either 5’ or 3’ untranslated region (UTR) [9]; global inhibition of translation by the eIF2alpha kinase IK2 in sporozoites [4]; as well as a role for the RNA binding protein Pumilio-2 (Puf2) in the sporozoite [1, 5, 6]. Puf2 has been shown to bind and control the translation of *pfs25* and *pfs28* in *P*. *falciparum* gametocytes [7]; such a role has not been identified in rodent malaria species. Instead, Puf2 in *P*. *berghei* and *P*. *yoelii* (a second rodent malaria model) controls the developmental progression from sporozoite to the so-called exo-erythrocytic liver stage form (EEF) [1, 5, 6]. In the absence of Puf2, sporozoites undergo morphological de-differentiation events seen only following liver cell infection, and lose infectivity [1]. Puf2 is therefore an essential developmental factor for salivary gland sporozoite (SGS) to liver stage transformation in the malaria parasite [1, 5, 6]. In *P*. *berghei* gene deletion mutants (Δ*puf2*) undergo progressive, and premature sporozoite to liver stage exoerythrocytic form (EEF) development in mosquito salivary glands over a period of nine days [1]. Morphologically, Δ*puf2* SGS are characterised by a rounding-up event which occurs only during the developmental program of the wildtype EEF and results from the breakdown of the inner membrane complex and subpellicular network.

How Puf2 controls these developmental changes, is unknown. A likely scenario involves the binding and translational regulation by Puf2 of certain mRNAs that drive the developmental progression that occurs following transmission of the parasite from the mosquito vector to the mammalian host.

Only two transcripts have been reported to be under post-transcriptional control in sporozoites involving unknown protein factors: *uis4* (up-regulated in infective sporozoites gene 4) [10–13] and *b9* [14]. While *b9* (a member of the 6-Cys family of surface proteins) has only been identified recently and appears not to be translated at all in *P*. *berghei* sporozoites [14], proteomic evidence in *P*. *yoelii* as well as *P*. *falciparum* attests its translation in sporozoites [15]. The expression data on *uis4* [16] is equally conflicting: many reports unambiguously detail UIS4 translation in salivary gland sporozoites (SGS) by Western and immunofluorescence analyses (IFA) [4, 12, 13, 16–19] while few find it not translated at all [5] or translationally repressed and hardly detectable [10]; the low levels of protein translation in the last study were shown to result from post-transcriptional silencing involving a form of recognition of the coding region of the gene, rather than involving 5’ or 3’ UTRs identified in transcripts encoding the *P*. *berghei* ookinete proteins P25 and P28 [8, 9]. In *P*. *berghei*, neither *b9* nor *uis4* mRNA levels are affected by the absence of Puf2 [1, 5]. The stability of *uis4* has been shown to rely on SAP1/SLARP [11, 12], a protein without known RNA binding domains, and inhabiting mRNPs that are clearly separate from those defined by Puf2 [6]. The majority of reports show UIS4 to localize to secretory organelles in sporozoites; the protein is then most likely discharged following definitive invasion of a liver cell by the sporozoite in the mammalian host in order to help establish for the first time and later maintain the parasitophorous vacuole membrane (PVM) which separates the parasite from the host cell cytoplasm [17, 20]. Throughout liver stage development, *uis4* is translated in order to maintain the parasite PV niche within the hepatocyte. UIS4 belongs to the ETRAMP family of proteins first characterised in *P*. *falciparum* [21, 22] and may only exist in rodent malaria species; it is unclear whether *P*. *falciparum* etramp10.3 (gene ids PF3D7_1016900 or PF10_0164) is a true ortholog. Although etramp10.3 also localises to the PVM, functionally it does not complement *P*. *yoelii* UIS4; like *uis4*, *etramp10*.*3* too is translated in sporozoites [18, 23].

Here we present a transgenic parasite line that expresses C-terminally GFP-tagged Puf2 (*puf2*::*gfp* line). The tagged RNA binding protein localises to cytoplasmic speckles in sporozoites. Using RNA-immunoprecipitation (Chromotek GFP-Trap_A approach), we find *uis4* mRNA bound by Puf2::GFP. Expressed at low levels in secretory vesicles of SGS in the wild type and the *puf2*::*gfp* line, a quantitative image analysis comparing wild type, *puf2*::*gfp* and a *puf2* gene deletion mutant [1] reveals a clear up-regulation of UIS4 protein expression in the absence of Puf2 and thus a regulatory role for Puf2 in the translation of this mRNA.

## Material and Methods

### Ethics statement for animal experimentation

All studies which involved mice were performed in strict accordance with the regulations of the Portuguese official Veterinary Directorate, which complies with the Portuguese Law (Portaria 1005/92). The Portuguese Experiments on Animal Act strictly comply with the European Guideline 86/609/EEC and follow the FELASA (Federation of European Laboratory Animal Science Associations) guidelines and recommendations concerning laboratory animal welfare. All animal experiments were approved by the Portuguese official veterinary department for welfare licensing and the Instituto de Medicina Molecular Animal Ethics Committee. All experiments were carried out using BALB/c mice (6–8 weeks of age; Charles River Laboratories International, Inc). Animal experiments performed in Leiden University Medical Center (LUMC, Leiden, The Netherlands) were approved by the Animal Experiments Committee of the Leiden University Medical Center (DEC 10099).

### Puf2 Plasmodium berghei mutant parasites

The mutant parasite line in which the *puf2* gene (PBANKA_071920) has been disrupted (PbA1267cl2; RMgm-516) has been described [1]. Transgenic parasites containing Puf2 tagged with a fluorescence reporter were generated by standard genetic modification technologies for *P*. *berghei* [24]. Parasites of the reference *P*. *berghei* ANKA (cl15cy1) line were transfected with a linear construct (pL1644) that introduces the fluorescent tag at the C-terminus of the endogenous gene by integration through single cross-over homologous recombination [24]. The *puf2* target region was PCR amplified with primer pair L3714 (AGATATCAAATTCCCTGAGCTAGCC) and L3715 (AGGATCCTGCCTCTAAATTATTAATAGCCC) and cloned into plasmid AB272 and transferred via Asp718I/EcoRV into vector AB106 (pL1326) containing the *eef1a-hdhfr* selectable marker cassette, resulting in the final plasmid pL1644. Transfected parasites were selected with pyrimethamine and cloned by limiting dilution as described [25] resulting in two parasite clones expressing C-terminally GFP-tagged *puf2* (1750cl3 and 1750cl4). The parasite line was genotyped and compared to the wild type reference line 259cl2 (WT PbGFPcon; RMgm-5) by PCR using the following primer combinations: g1338 (TTAAATACGAAGACAATC) and g0408 (GTATGTTGCATCACCTTC) for 5’ integration of the plasmid; g0359 (GTTTTCCCAGTCACG-ACGTTG) and g1311 (AAAAAATTTGAAGC-CGCC) for 3’ integration of the plasmid; g1338 and g1335 (TAAATGTCTGTGTTGTCC) for the wild type *puf2* gene; g1339 (ACGAATTTAGATATTTCC) and g1340 (ATCATTCTTCTCATATAC) for the human dhfr selection marker; g0084 (TGATGGTTTACAATCACC) and g0085 (TTCTTCCTGCATCTCCTC) for a control PCR. To confirm exclusive mutant allele transcription, following reverse transcription, PCR of *puf2*::*gfp* was performed with primer pairs g1310 (GACATTCCTGAGGGAAATG) and g0408 (GTATGTTGCATCACCTTC), or g1310 and g1311 (AAAAAATTTGAAGCCGCC) for the wild type transcript. Input controls were *hsp70* [primers g0258 (AAAAGCAAAGCCAAACTTACC)and g0259 (GGATGGGGTTGTTCTATTACC)] and *18S ribosomal RNA* [primers PbA18SFw (AAGCATTAAATAAAGCGAATACATCCTTAC) and PbA18SRev (GGAGATTGGTTTTGACGTTTATGTG)].

### *Anopheles stephensi* mosquito infection and parasite development

*A*. *stephensi* were bred at the insectary of the Instituto de Medicina Molecular (IMM). For mosquito infection, female BALB/c mice were intraperitoneally injected with *P*. *berghei* wild type, *puf2*::*gfp* or Δ*puf2* mutant lines. Mosquitoes were allowed to feed on anaesthetized mice for 25 minutes on two consecutive days and sporozoites collected 18, 22 and 27 days later. For transmission experiments naïve BALB/c mice were exposed to 10 infected *puf2*::*gfp* (1750cl3 or 1750cl4) infected mosquitoes for 30 minutes; 3 mice were used per parasite clone. Parasitemias were followed daily by counting Giemsa smears from a drop of tail blood.

### RNA-immunoprecipitation (RNA-IP) experiments

Puf2::GFP RNA-IP was performed with pooled day 22 post-infection sporozoites using a total of 756 infected female mosquitoes following protocols established for Puf2 RNA-IP in *P*. *falciparum* [7] and DOZI and CITH in *P*. *berghei* [2, 3, 8, 26, 27]. Briefly, mosquito midguts and salivary glands were dissected in PBS supplemented with protease inhibitors (ROCHE), grinded with a pestle and passed through a 70 μM filter. The sporozoite material was centrifuged at maximum speed for 15 minutes at 4°C. The pellet fraction was used for IP with the GFP-Trap^®^_A Kit (Chromotek) following the manufacturer’s instructions. Total RNA from IP eluates was isolated with TRIZOL, reverse transcribed with random hexamers and oligo d(T) oligonucleotides, and analysed by PCR using the following primer pairs: g0444 (CCAAACCAAG-CGATCATACATACAG) and g0445 (CTTCACCCACTAAATCGCTTAATTC) for *uis4*; g0814 (AAACTTTATTCAGCACCTGAAC) and 0815 (CGGTAAAAATATGTCACCAGC) for *uis1/ik2*; g0430 (ACAGAGGAATGGTCTCAATG) and g0535 (CATTTATCCATTTTACAAATTTCAG) for *csp*; g0151 (AATCGAAGCAAAAGGTGG) and g0152 (AATTTATATGGGAGCTCC) for *slarp*; g1042 (TTCCATAGCAAACTTGTG) and g1092 (CATGCGTGCATATATAGAACG) for *pbanka_100250*; g1065 (ACTCATAGATTCATATTTATAC) and g1094 (CCGTGATGAATATTCTGTTCAAC) for *pbanka_123370*; and g0432 (AACATTCACTCCATTCTTCC) and g0433 (CATGTTATTCCAATGCTCAC) for *trap*.

### Immunofluorescence assays

Salivary glands infected with *puf2*::*gfp* and Δ*puf2* parasites were collected in 1xPBS and smashed with a pestle to release the sporozoites. The sporozoites were allowed to adhere to glass slides, air-dried, fixed with 4% PFA/PBS for 10 minutes, and washed 3x5 minutes with 1xPBS. Sporozoites were permeabilized with 0.1% Triton-X100/PBS for 10 minutes at room temperature (RT) followed by a single 5 minutes wash with 1xPBS. Slides were blocked with 1% BSA/PBS for 1 hour at RT, incubated with the primary antibody in 1% BSA/PBS for 1 hour at RT, washed 3x5 minutes with 1xPBS, incubated with the conjugated secondary antibody for 1 hour at RT (protected from light), washed 3x5 minutes with 1xPBS, and incubated with 1 μg/mL Hoechst/PBS for 1 minute at RT (protected from light). The slides were then washed once in 1xPBS, mounted with Fluoromount-G (SouthernBiotech) and stored at 4°C until analysed using a wide field fluorescence Leica DM5000B microscope. The primary antibodies used were a rabbit polyclonal anti-GFP (1:500; Abcam # ab6556), goat polyclonal anti-UIS4 (1:1000; SICGEN # AB0042-200), or a mouse monoclonal anti-CSP (3D11; 5 μg/mL). The secondary antibodies used were goat anti-rabbit Alexa 488 (1:400; Jackson # 111-545-003), goat anti-rabbit Alexa Fluor 594 (1:400; Jackson # 111-586-047), rabbit anti-goat Alexa Fluor 594 (1:400; Invitrogen A-11080) and goat anti-mouse Cy3 (1:250; Jackson # 115-166-003).

### ImageJ quantification of UIS4 expression intensity

Fluorescence images were captured with a Leica DM5000B fluorescence microscope (objective 100x), with the following settings: DAPI 50.6 ms, red channel 575 ms for UIS4 and DIC 2.01 ms. Fluorescence intensity quantifications were performed with ImageJ software on 8-bit black and white pictures. The shape of each sporozoite was outlined by freehand selection and the fluorescence intensity determined using the ImageJ histogram function; the exact same outline was used on an adjacent area of the image to provide a background value.

## Results

Puf2 is an essential developmental factor during salivary gland sporozoite (SGS) to liver stage transformation in the malaria parasite [1, 5, 6]. In *P*. *berghei* gene deletion mutants Δ*puf2* undergo progressive, and premature sporozoite to liver stage exoerythrocytic form (EEF) development in mosquito salivary glands over a period of nine days [1]. Morphologically, Δ*puf2* SGS are characterised by a rounding-up event which occurs only during the developmental program of the wildtype EEF and results from the breakdown of the inner membrane complex and subpellicular network.

### GFP-tagging of Puf2 maintains wildtype growth and transmission

**Fig 1:**
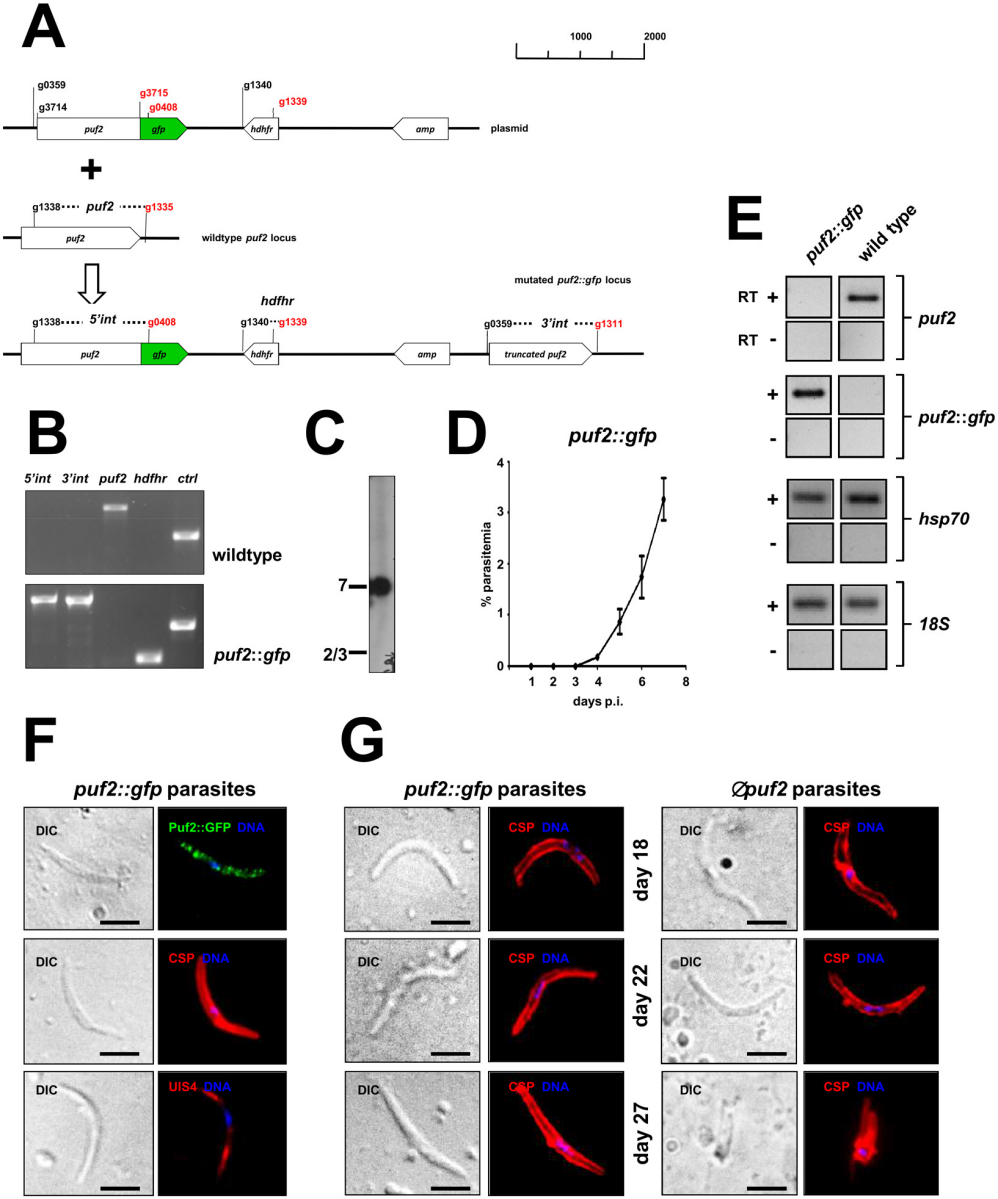
PUF2::GFP behave like wildtype parasites and maintain a latent salivary gland parasite population. **(A)** Schematic of genetic modification of the *puf2* locus. Shown are position of primers (red: reverse) and genotyping PCRs (italics) as shown in B. **(B)** PCR genotyping of *puf2*::*gfp* parasite clone compared to wild type; from left to right are shown: 5’ and 3’ integration sites of the plasmid construct; wildtype *puf2* locus; human *dhfr* selection marker; a control reaction. See A for position of primer pairs. **(C)** Field inversion gel electrophoresis followed by Southern blot analysis detects correct integration of the plasmid construct into chromosome 7. **(D)** Parasitemia development of *puf2*::*gfp* (n = 6) in Balb/C mice following mosquito bite (10 mosquitoes per mouse were allowed to feed for 30 minutes). days p.i. = days post mosquito infection. Mean ± s.d. **(E)** RT-PCR performed on RNA isolated from the *puf2*::*gfp* parasite line sporozoites and wild type (PbA259) sporozoites from days 20/21 post-mosquito infection confirm transcription of *puf2*::*gfp* mRNA in the *puf2*::*gfp* parasites and not the wildtype, untagged gene. *hsp70* and *18S* rRNA were amplified as control genes. **(F)** Immunofluorescence assay (IFA) of salivary gland sporozoites from days 21/22 post-mosquito infection. Rabbit anti-GFP antibody ab6556 (Abcam), mouse anti-CSP (3D11), or goat anti-UIS4 antibody (SICGEN) were used. Scale bars = 5μm. **(G)** IFA of *puf2*::*gfp* and *puf2* sporozoites at days 18, 22 and 27 post-mosquito infection; mouse anti-CSP (3D11) was used. Note the morphological change (rounding up) of day 27 *puf2* parasites. Scale bars = 5 μm.

The presence of an evolutionarily conserved RNA binding domain (the Pumilio homology domain) in *P*. *berghei* Puf2 [1] suggested that this protein might control the stability or translational efficiency of certain, yet unknown, transcripts that when deregulated resulted in the observed transformation event. In order to address the question which mRNAs might be bound by Puf2 in the *P*. *berghei* sporozoite, we generated the GFP-tagged line *puf2*::*gfp* (Fig 1A to 1C) to be used in RNA immunoprecipitation experiments. The two *puf2*::*gfp* clones examined behaved like wild type parasites during re-infection of naïve mice. Following mosquito bite, we found the pre-patent period (time taken between infection and appearance of blood stage parasitemia visible in a Giemsa smear by microscopy) to be in the wild type range with parasites detectable in the blood at day 4 (Fig 1D) while Δ*puf2* sporozoites fail to establish blood stage infections entirely [1, 5, 6]. Tagging of the endogenous allele in the haploid *P*. *berghei* parasite ensures wild type transcriptional control of *puf2*::*gfp* and results in exclusive expression of the tagged allele (Fig 1E). Puf2::GFP protein localised to cytoplasmic foci in SGS indicating the presence of localized messenger ribonucleoproteins (mRNP) (Fig 1F). These *puf2*::*gfp* parasites expressed the major surface marker circumsporozoite protein (CSP) as expected for this developmental stage, as well as UIS4 as reported [4, 12, 13, 16–19] (Fig 1F). Consistent with the observed infectivity of *puf2*::*gfp* parasites (Fig 1D), these mutants maintained a slender, wild type shape from day 18 through to day 27 post-infection. On the other hand, 85% (n = 139) of day 27 Δ*puf2* mutants showed clear signs of transformation and loss of sporozoite-like morphology (Fig 1G).

### Puf2::GFP binds uis4 mRNA

**Fig 2:**
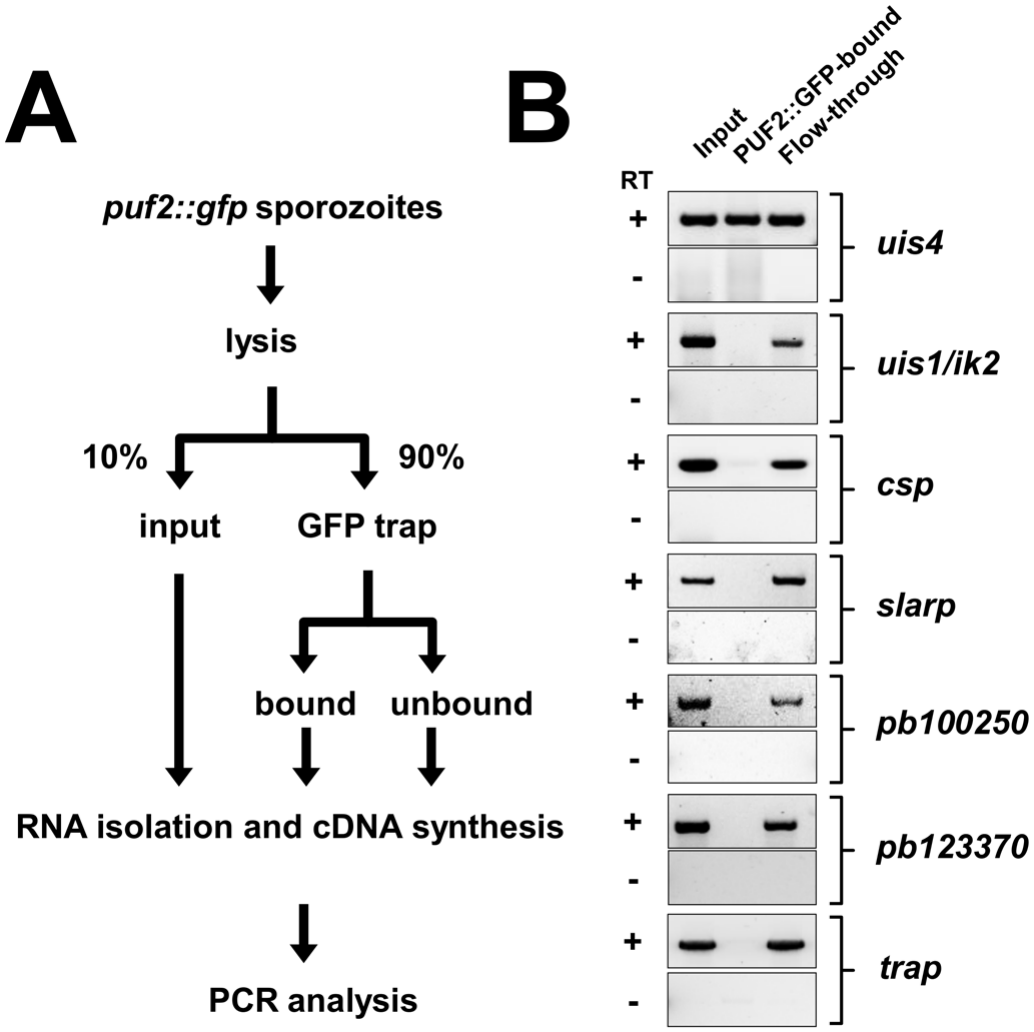
RNA immunoprecipitation of PUF2::GFP from *puf2*::*gfp* salivary gland sporozoites from day 21 post mosquito infection reveals binding of *uis4* by PUF2::GFP. **(A)** Schematic of the GFP-Trap_A Kit (Chromotek) IP protocol used. (**B)** RT-PCR performed on RNA isolated from the input and IP eluates as outlined in A to verify the presence or absence of *uis4* and control genes in IP eluates. The material used for the input RT-PCR represents 6.7% of bound as well as unbound samples. RT+ and RT- indicate cDNA synthesis set-up in the presence (+) or absence (-) of Reverse Transcriptase.

The transmission experiment (Fig 1D) and IFA data (Fig 1F and 1G) showed that the C-terminal GFP-tag did not alter the functionality of Puf2. The mutant behaved like wild type and maintained an infectious, morphologically normal parasite population. We therefore attempted the isolation of Puf2::GFP-bound transcripts in sporozoites by RNA immunoprecipitation (RIP) (Fig 2A) following methods employed successfully in *P*. *falciparum* [7] and *P*. *berghei* [2, 3, 8]. This was followed by isolation of total RNA and detection of selected transcripts by RT-PCR in the input, bound and flow-through fractions to identify potential Puf2 bound transcripts. Only *uis4* and *b9* had been reported to be translationally repressed in *P*. *berghei* sporozoites [10, 14, 28] and thus represented key candidate target mRNAs in the RT-PCR. We included a selection of six transcripts known to be translated and which thus would serve as controls (Fig 2B). *uis4*, *trap*, *ik2*, *slarp*, *csp*, *pbanka_100250*, *pbanka_123370* were amplified in the input and flow-through fractions; only *uis4* could also be amplified from the cDNA generated from the bound fraction showing that it was bound by Puf2::GFP. The signal for *uis4* in the flow-through fraction could represent transcript lost during the RIP procedure or mRNA not associated with the Puf2 complex. We were unable to amplify *b9* from the input material, and thus could not determine whether this gene represents a Puf2 target.

### Absence of Puf2 results in upregulation of UIS4 protein translation

We and others had previously shown that *uis4* mRNA levels are not affected by the absence of Puf2 [1, 5]. Binding of *uis4* by Puf2::GFP or a complex defined by Puf2::GFP therefore suggested that Puf2 controls the translational efficiency of this mRNA rather than its stability. In *P*. *falciparum* gametocytes, *pfs25* and *pfs28* are bound by Puf2 resulting in the translational repression of both mRNAs prior to parasite transmission [7]. In higher eukaryotes, members of the Pumilio protein family act as post-transcriptional repressors that when bound to a target mRNA affect its stability, localization or translation [29–31].

**Fig 3:**
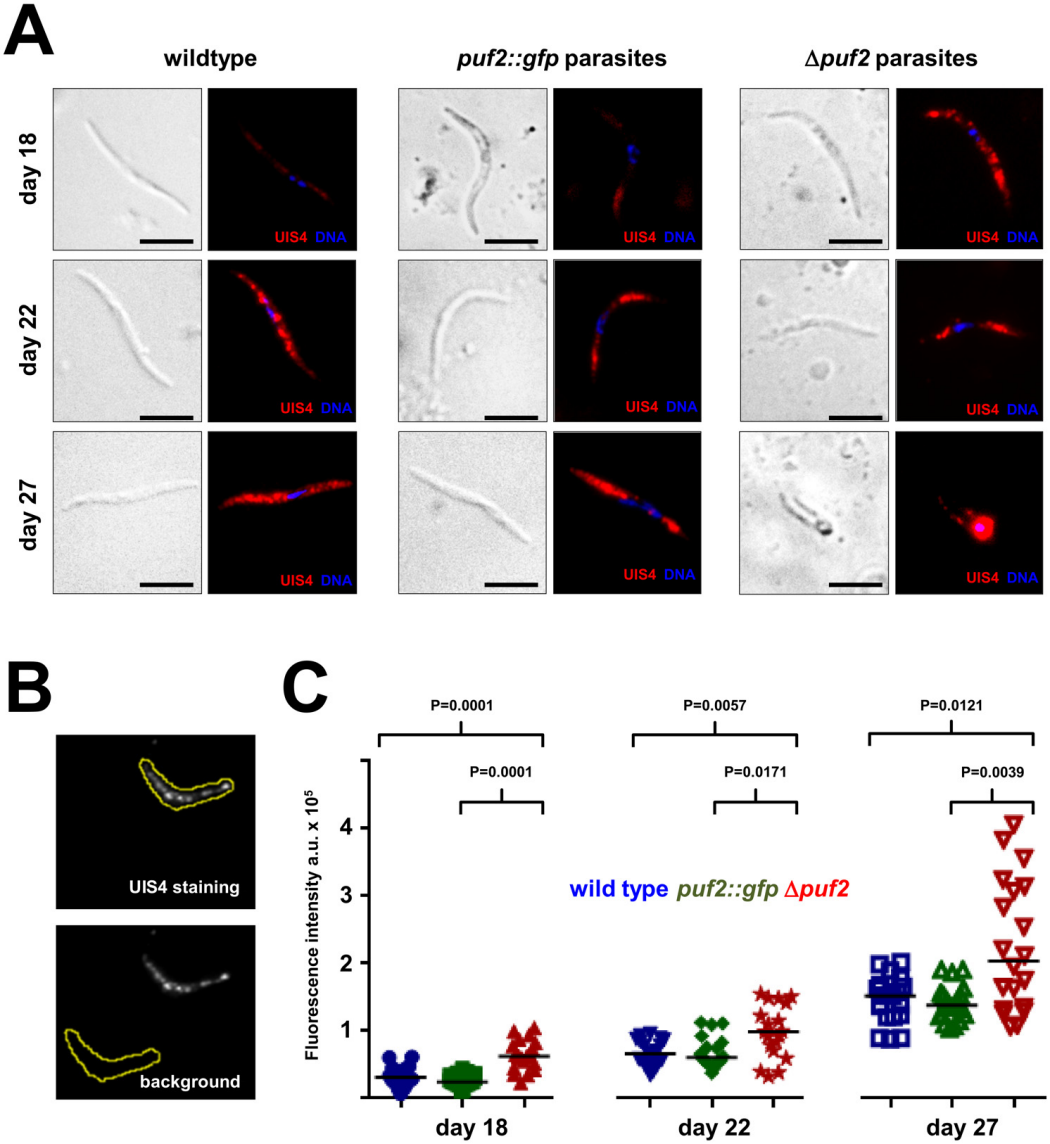
UIS4 protein expression is upregulated in *puf2* salivary gland sporozoites. **(A)** UIS4 was detected by immunofluorescence assay with a goat anti-UIS4 antibody (SICGEN) in salivary gland sporozoites from days 18, 22 and 27 post-mosquito infection in the indicated parasite lines. Note the change in morphology of the *puf2* sporozoite. Scale bars = 5 μm. **(B)** Fluorescence images representing the quantification method used with ImageJ software. UIS4 immunofluorescence of a *puf2* sporozoite at day 22 post-mosquito infection is shown. The shape of the sporozoite was outlined; the same shape was placed on a neutral area of the image to obtain a background value. Fluorescence intensities of the sporozoite and background were determined with ImageJ software as explained in the Material and Methods. **(C)** Scatter plot representation of the UIS4 fluorescence intensity measurements (arbitrary units a.u.) are shown (n = 20 for each timepoint and parasite line). P-values were obtained by Mann-Whitney test.

In order to determine whether Puf2 affected the translational efficiency of *uis4*, we performed immunofluorescence assays for UIS4 with a specific antibody using SGS isolated on three different days (18, 22 and 27) following mosquito infection. All wild type, *puf2*::*gfp* and Δ*puf2* parasites expressed UIS4 at all time points examined (Fig 3A). The staining appeared the weakest in wild type and *puf2*::*gfp* lines at day 18, and increased over the next 9 days of infection in all three lines and was strongest in transformed, EEF-like Δ*puf2* parasites at day 27. We next quantified the UIS4-staining intensities in *puf2*::*gfp*, wild type and knock-out Δ*puf2* sporozoites with ImageJ (Fig 3B). The intensities of UIS4-staining in wild type and *puf2*::*gfp* lines were similar in each of the three time points and increased continuously from day 18 to day 27 in both lines (Fig 3C); compared to staining intensities at day 18, the fluorescence increased to approximately 5-fold in wild type and the *puf2*::*gfp* line at day 27. The similar results for UIS4 expression in wild type and *puf2*::*gfp* lines is consistent with the mutant behaving like the wild type during our transmission experiments. The Δ*puf2* line, on the other hand, deviated significantly from the other two lines: at day 18 Δ*puf2* parasites showed already a two-fold increase in UIS4-staining intensity (Fig 3C) and remained elevated throughout the observation period. The absence of Puf2 therefore resulted in increased expression levels of UIS4.

## Discussion

UIS4 is critical for liver stage development of the rodent malaria parasite with a role in growth and maintenance of the PVM but redundant for liver cell invasion [17, 20]. Here we show that *uis4* mRNA is bound directly by Puf2::GFP or a complex containing this protein in SGS. In the absence of Puf2, controlled timing of UIS4 translation is lost and translation increased in the salivary gland sporozoite. Our data could mean that some *uis4* mRNA is Puf2-bound and thus translationally silenced and stored, while a second pool is translated and trafficked to secretory vesicles. This would provide UIS4 protein to be secreted during productive invasion of the host hepatocyte to help establish the very first PVM—the time from mosquito bite to liver cell invasion can be as little as eleven minutes [32]. *uis4* mRNA on the other hand could provide the template for protein translation from the stored pool until activation of the EEF transcriptional profile.

The bulk of UIS4 protein is expected to be produced after the sporozoite has invaded the mammalian host hepatocyte. In Δ*puf2* parasites however there is a clear premature translation of *uis4* mRNA (data shown here). Similarly, incubation of salivary gland sporozoites at 37°C results in an upregulation of UIS4 protein in wild type cells within half an hour; in Δ*ik2* mutants (that develop precociously similarly to the Δ*puf2* parasite) UIS4 protein levels remain unchanged throughout this period and in the beginning are as high as those reached by the wild type after 30 minutes [4]. Together with the observation that the precocious expression of a UIS4::mCherry transgene results in a loss of parasite infectivity with reduced liver loads [10] the data explain the reduced infectivity and development of Δ*puf2* parasites [1] and the Δ*ik2* mutant sporozoite line [4]; both mutants fail to infect by mosquito bite. Whether the Δ*ik2* and Δ*puf2* are linked is unclear. *ik2* is not bound by Puf2 but in the absence of *puf2* is downregulated [1]. Like suggested already for *P*. *falciparum* gametocytes [7], multiple pathways may secure tight translational control during gametocyte as well as sporozoite transmission.

Whether Puf2 recruits additional proteins to a larger mRNP in order to suppress *uis4* translation is unknown, but likely. In many organisms Pumilio is associated with the inhibition of translation of specific target mRNAs, usually by repressing translation or enhancing mRNA decay [30], and depending on the specific function, Puf proteins can have a variety of partners: these include Nanos (nos) [33–36], Nanos-Brat [37], Nanos-Gemin3 [38], Nanos-CPEB [39], CPEB [40], argonaute-eEF1A [41], and the CCR4-POP2-NOT deadenylase complex [42, 43]. The current data do not support that Puf2 and SAP1/SLARP act in concert. First of all, Puf2 and SAP1 are present in separate mRNP granules in salivary glands sporozoites of *P*. *yoelii* [6]. Secondly, the stability of *uis4* mRNA does not require Puf2 [1, 5] while the absence of SAP1/SLARP results in a 20-fold reduction in mRNA levels; although left with only 5% of wild type mRNA levels the protein is still detectable [12]. The loss of *uis4* in *P*. *yoelii* Δ*sap1/slarp* sporozoites is due to mRNA degradation, and here no UIS4 protein is detectable at all [13]. Our results show that Puf2 acts as a translational regulator of *uis4* mRNA translation, rather than protecting the transcript from degradation like SAP1/SLARP. Using *P*. *berghei* lines expressing transgenes, Silvie *et al*. had shown that the open reading frame (ORF) of *uis4* contains an unknown, but crucial element for the translational repression of a fluorescently tagged *uis4* transgene [10]. A single Pumilio-like recognition motif (UGUAAACA) is present in the *P*. *berghei uis4* mRNA at nucleotide positions 284–292 of the ORF (UGUAAACA). The related UGUAHAUA (H = A/C/U) motif is recognized by human Pum1 [44], *Drosophila* Pumilio [45], yeast Puf3 [46] and most importantly by *P*. *falciparum* Puf2 [7]. In the human malaria parasite *pfs25* and *pfs28* are directly bound by Puf2 and kept translationally silent prior to transmission; in the absence of Puf2 the translation levels of these proteins are upregulated. Whether this *uis4* motif truly recruits *P*. *berghei* Puf2 and thus regulates translational of a mRNA subpopulation is unknown.

*uis4* is unlikely to be the sole target for Puf2-mediated translational control in *P*. *berghei*. The profound morphological changes experienced in its absence suggest that key developmental factors are being regulated by this RNA binding protein. In *P*. *berghei*, the current hypothesis is that Puf2 regulates the translation of one or several key factor(s) that once translated play a role in the developmental progression from salivary gland sporozoites to early liver stage forms [1, 5, 6]. The identity of such key factors is, however, unknown. The Puf2-dependent translational regulation of *uis4* represents an attractive mechanistic model that could act on a larger pool of transcripts, some of which encode such developmental factors.
